# PW03-022 – Neutrophilic skin disease and inflammation

**DOI:** 10.1186/1546-0096-11-S1-A248

**Published:** 2013-11-08

**Authors:** K Webb, C Hlela, C Scott

**Affiliations:** 1 Paediatric Rheumatology, Cape Town, South Africa; 2 University of Cape Town, Cape Town, South Africa

## Introduction

Robert Sweet first described a syndrome with a painful, erythematous nodular plaques, neutrophilic dermal infiltrates, fevers and peripheral neutrophilia. This cluster of syndromes became known as Sweet’s syndrome. There have been many published cases in children of neutrophilic dermatoses and fever which are labeled as Sweet’s syndrome. Recently, however, neutrophilic dermatoses have been associated with some autoimmune and autoinflammatory diseases .

## Objectives

To present 3 cases of children with differing manifestations of neutrophilic skin disease and systemic inflammation and postulate on different possible autoinflammatory pathophysiological causes.

## Methods

Retrospective case review was conducted.

## Results

Patient 1 is a 3 year old child was referred from dermatology with recurrent intermittent episodes of annular erythematous lesions,arthralgias, fevers, red eyes and irritability. Clinically she had episcleritis, conjunctivitis, fevers, failure to thrive, markedly elevated inflammatory markers and a microcytic anaemia. Skin lesions were painful,annular erythematous plaques with cutis laxa. Histology demonstrated a neutrophilic dermatosis which responded to a course of steroids.

Patient 2 is a 6 month old girl who presented in 2012 with erythematous, nodular plaques on trunk, arms, legs and face since 6 weeks of age. These were accompanied by fever, raised white cells and raised inflammatory markers. She had been steroid dependant since 6 weeks of age. Histology showed a leukocytoclastic neutrophilic lobular panniculitis and dermatitis.

Patient 3 is a child with panniculitis (neutrophilic on histology) raised inflammatory markers, arthritis and lipodystrophy. She also presented with hepatitis, myositis, nephritis and macrophage activation syndrome. She was diagnosed with chronic atypical neutrophilic dermatosis, lipodystrophy and elevated temperature (CANDLE) syndrome, a recently described autoinflammatory condition.

## Conclusion

We review the recent evidence that autoinflammation may play a role in neutrophilic skin diseases, including recent reports of therapy with IL1 inhibition.We propose that some conditions previously labelled Sweet’s Syndrome could possibly represent a manifestation of autoinflammatory conditions.

## Disclosure of interest

None declared

